# Multicenter trial of motion analysis for injury risk prediction: lessons learned from prospective longitudinal large cohort combined biomechanical - epidemiological studies

**DOI:** 10.1590/bjpt-rbf.2014.0121

**Published:** 2015-10-06

**Authors:** Timothy E. Hewett, Benjamin Roewer, Kevin Ford, Greg Myer

**Affiliations:** 1 Mayo Clinic, Rochester, MN, USA; 2 Sports Health & Performance Institute, The Ohio State University, Columbus, OH, USA; 3 Departments of Physiology and Cell Biology, Orthopaedic Surgery, Family Medicine and Biomedical Engineering, The Ohio State University, Columbus, OH, USA; 4 Division of Sports Medicine, Cincinnati Children's Hospital Medical Center, Cincinnati, OH, USA; 5 Department of Pediatrics and Orthopaedic Surgery, College of Medicine, University of Cincinnati, Cincinnati, OH, USA; 6 Department of Physical Therapy, School of Health Sciences, High Point University, High Point, USA

**Keywords:** ACL, adolescents, knee injuries, drop vertical jump, prevention

## Abstract

Our biodynamics laboratory group has conducted large cohort biomechanical-epidemiological studies targeted at identifying the complex interactions among biomechanical, biological, hormonal, and psychosocial factors that lead to increased risk of anterior cruciate ligament (ACL) injuries. The findings from our studies have revealed highly sensitive and specific predictors for ACL injury. Despite the high incidence of ACL injuries among young athletes, larger cohorts are needed to reveal the underlying mechanistic causes of increased risk for ACL injury. In the current study, we have outlined key factors that contribute to the overall success of multicenter, biomechanical-epidemiological investigations designed to test a larger number of athletes who otherwise could not be recruited, screened, or tested at a single institution. Twenty-five female volleyball players were recruited from a single high school team and tested at three biodynamics laboratories. All athletes underwent three-dimensional motion capture analysis of a drop vertical jump task. Kinematic and kinetic variables were compared within and among laboratories. Reliability of peak kinematic variables was consistently rated good-to-excellent. Reliability of peak kinetic variables was consistently rated goodto-excellent within sites, but greater variability was observed between sites. Variables measured in the sagittal plane were typically more reliable than variables measured in the coronal and transverse planes. This study documents the reliability of biomechanical variables that are key to identification of ACL injury mechanisms and of athletes at high risk. These findings indicate the feasibility of executing multicenter, biomechanical investigations that can yield more robust, reliable, and generalizable findings across larger cohorts of athletes.

## Introduction

 Our multicenter, multidisciplinary research group has conducted several collaborative, multi-institutional studies that included reliability comparisons of biomechanical and neuromuscular data from three different sites - the Biodynamics Laboratories at Cincinnati Children's Hospital (CCH or Site A), The Ohio State University (OSU or Site B) and the University of Kentucky (UK or Site C) - using identical data collection, reduction techniques, data processing methods, and data analyses. Reliability studies of this kind, as with any such impactful study, are important for the establishment of widespread generalizability, reliability, reproducibility, and acceptability of multicenter collected data. The authors have previously tested and measured the longitudinal reliability and validity of all of the data collected during testing from one site at Cincinnati Children's Hospital (CCH)1. In order to conduct a proper measurement of the validity of these prospective cohort study findings, we track injuries prospectively so that we can effectively use these data for widespread injury risk assessment.

### Long-term objectives of multicenter biomechanical-epidemiologic studies

The primary objectives of our Multicenter Biomechanical-Epidemiologic studies are to determine how individuals become more susceptible to injury, prospectively identifying those athletes who are more susceptible to injury and to determine the underlying mechanistic cause(s) of increased risk at the biomechanical level and to optimize the effectiveness of treatments designed to prevent these injuries. Towards these goals, we test hypotheses related to multiple biomechanical variables: lower extremity bone length and body mass maturational stage; neuromuscular performance; whole limb and whole body posture; trunk, hip, and knee joint loading; and injury risk in subsets of athletes.

Our research group has many ongoing studies and our research interests and activities can be broken down into three areas of study: 1) Mechanistic Studies using *In Sim* approaches that combine multiple *in vivo*, *in vitro*, cadaveric, computer modeling, and animal model approaches to answer the most pertinent questions in our field; 2) High-Risk Individuals Studies using evidence-based medicine (EBM) datasets to determine which athletes are at increased risk for anterior cruciate ligament (ACL) injuries; and 3) Preventive Studies using Randomized Controlled Trial (RCT) designs to determine which interventions decrease risk for ACL injuries in large cohort populations. We also employ dual identifying and preventive studies using EBM techniques and datasets and RCT designs to determine which interventions are most efficacious in specific athletes, both individuals and groups, which are at increased risk for ACL injuries.

### School and community-based research partnerships

Over the past two decades, we have collaborated with large public geographic county-based schools. Our primary methods of recruitment were data-driven, state-of-the-art presentations and cutting-edge tools used in areas of particular interest, as they fit nicely into school administrators' overall objectives. These school administrators included superintendents, athletic directors, principals, teachers, and coaches.

Our primary objectives with these studies have been to collaborate with school administrators, coaches, students, and parents to gain support for our coupled Biomechanical-Epidemiologic studies, to design and develop screening protocols to identify high-risk athletes who demonstrate identifiable neuromuscular control deficits that put them at risk, and to develop and implement neuromuscular training interventions to decrease injury risk. Our overall objectives for the current theoretical construct include understanding the United States' National Institute of Health (NIH) funding opportunities; technology options for recruiting and retention; tracking options for study/trial management; tips to help avoid time delays in study implementation and to detail technical research methods and tool options and methods.

### Multicenter trial of motion analysis for injury risk prediction in school settings: experimental approach & methods

The goals of our experimental approach are to determine the injury risk predictive role of specific factors such as trunk, hip, and knee position; strength and muscle recruitment at the hip and knee; hip and knee load and neuromuscular control, i.e. adolescent growth and increases in tibia and femur length; and body mass in athletes. Our group utilizes what we term a 'Top-down & Bottom-Up' administrative approach to implement these studies. We begin our explorations of the school systems with school administrators including superintendents, athletic directors, and principals. We then contact coaches, athletic trainers (ATs), and teachers to get their full support with these research studies.

### Overall couple biomechanical-epidemiologic approach

For over a decade, we have conducted prospective longitudinal large cohort combined biomechanical-epidemiological studies. ACL injury risk has proven to be a complex, multi-faceted problem that involves biomechanical, biological, hormonal, and psychosocial factors. We have tested the hypothesis that measures related to dynamic lower extremity valgus will prove predictive of ACL injury risk in high-risk female athletes. For example, Myer et al.^2^ demonstrated that female athletes with increased knee recurvatum had significantly increased risk of ACL injury. We also demonstrated that increased lateral trunk displacement following quick-release perturbation was indicative of increased risk of anterior cruciate ligament injury in females, but not males^3^. In another longitudinal study, we showed that there were changes in both knee joint and general joint laxity with growth and development that differ between females and males^4^. We have also published our examinations of the contributions of both coronal and sagittal plane kinematics to dynamic stability of the knee and the reliability of our 3D Motion Analysis measures^1^.

**Figure 1.**
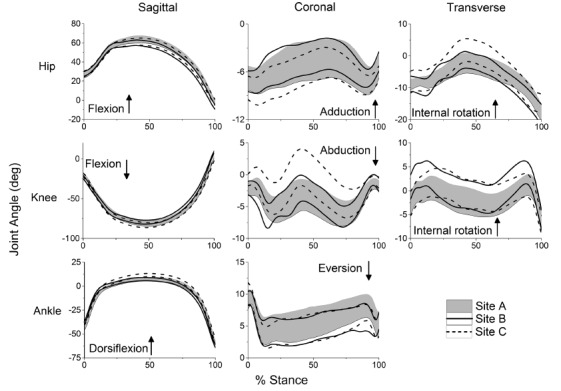
Time series for hip, knee and ankle angles time-normalized to 100% of stance. Lines represent upper and lower 95% confidence interval bounds.

**Figure 2.**
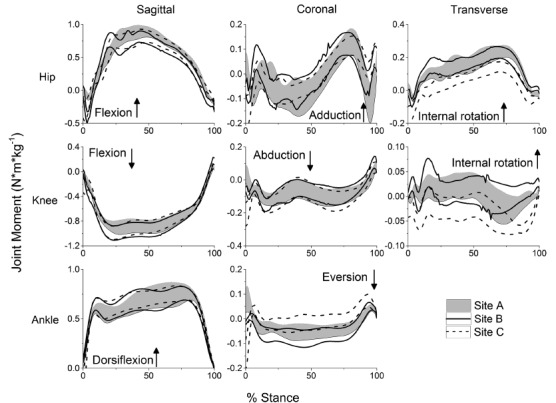
Time series for hip, knee and ankle moments time-normalized to 100% of stance. Lines represent upper and lower 95%

We utilized a prospective longitudinal design in county-based school-sponsored soccer, volleyball, and basketball teams from multiple school systems that we recruited, tested, and tracked. Female and male subjects from high school and junior high school were screened prior to the start of multiple consecutive soccer, volleyball, and basketball seasons. Graphs depicting the standardized means with standard deviations from the demographic variables of the subject population are shown in Figures 1 and 2. 

 confidence interval bounds.

### Subject recruiting

For over two decades, our research group has recruited hundreds of high school teams that have yielded thousands of soccer, volleyball, and basketball players who were pre-screened, trained, and post-screened^5^. We faced multiple challenges recruiting soccer players at junior high schools in the public county school systems. Some of the junior high schools did not have organized teams. We have addressed this challenge via recruitment of teams outside the county system (e.g. non-public, parochial schools located in the same county geographic area) in order to fill in all of our randomized blocks. In addition, we captured those junior high school athletes who went on to play high school soccer, volleyball, and basketball within the county school system.

### Multicenter reliability biostatistical analyses: biomechanical data

Careful biostatistical analysis should be performed by experienced biostatisticians and biomechanical profiles should be created for each of the screening movements. Standardized values should be used, as the variables are on different scales with varying mean values. Due to underlying normality assumption, it was necessary to transform some relevant variables to the log_e_ scale. In addition, the correlations between variables needed to be accounted for, in particular maximum hip and knee angles and moments. We continue to examine biostatistical models and will check them against data to determine any emergent predictive profiles. Comparison of mean variables, with and without adjustment for potential confounders, should also be the focus of future analyses.

## Method

**Figure 3.**
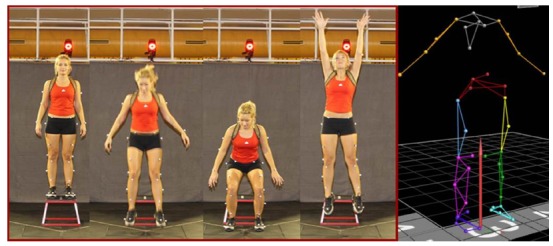
Example of the marker set used in this study as seen during the drop vertical jump (DVJ) task (left). Computerized representation of the marker used in this study (right).

A total of 25 volleyball players from a single High School team were screened in August of 2011. Testing was completed over two weeks in that month. All subjects provided informed consent approved by the institutional review boards at the University of Cincinnati, Cincinnati, OH, USA, The Ohio State University, Columbus, OH, USA, and University of Kentucky, Lexington, KY, USA (approval number 2011H0075, 022-11, and 08-0573, respectively). Each subject was instrumented with fortythree (43) 9-mm retroreflective markers by a different research assistant at each laboratory site. Markers were placed over the spinous process of C7; the midpoint between the suprasternal notch and the second costal notch of the sternum; the L5-S1 spinal junction; left posterior superior iliac spine; and bilaterally on the shoulders, upper arms, elbows, wrists, anterior superior iliac spines (ASIS), greater trochanters, mid thighs, medial and lateral knee joint lines, tibial tubercles, distal and lateral shanks (i.e. lower part of leg), and medial and lateral ankles, as shown in Figure 3. Subjects wore a small backpack outfitted with three non-collinearly placed markers. Each subject wore the same make and model of shoes with markers permanently affixed to the heels, dorsal surface of the mid foot, fifth metatarsal, and superior surface of the toe. Marker position and force data were collected using commonly accepted motion analysis techniques. A static trial was recorded with the subject standing in a neutral anatomical alignment with his/her foot placement standardized to the laboratory coordinate system. Subjects performed three drop vertical jump (DVJ) maneuvers from a height of 31 cm. Their feet were initially positioned 35 cm apart, and the subjects were instructed to drop off the plyometric box and perform a maximal vertical jump, reaching up towards a target placed directly overhead of the force plates at the height of their maximum vertical jump, as seen in Figure 3. 

Data collected at each of the three sites were obtained using similar equipment and sampling frequencies. Different personnel collected data and instructed subjects at each site. A detailed equipment list is specified in Table 1. 

**Table 1.** Summary of motion capture data collection equipment and techniques used at each data collection site.

**Site**	**A**	**B**	**C**
Camera System	10-camera Motion Analysis	8-camera Vicon	18-camera Motion Analysis
Force platforms	AMTI (600 x 900mm)	Bertec (300 x 600mm)	Bertec (600 x 900mm)
Sampling frequency (marker / force)	240 Hz/1200 Hz	240 Hz/1200 Hz	200 Hz/1000 Hz
Data processing software	Motion Analysis Cortex	Vicon Nexus	Motion Analysis Cortex

A, B & C: Cincinnati Children's Hospital, The Ohio State University, and the University of Kentucky, respectively.

We had internally verified that 3-D marker position data collected simultaneously on Vicon (VICON, Oxford Metrics Ltd., London, UK) and Motion Analysis (Motion Analysis Corp., Santa Rosa, CA, USA) camera systems using the same 240 Hz sampling frequency and the same data reduction techniques yielded no significant differences in computed joint angles^6^. Data collection procedures were developed and optimized in order to collect reliable and valid data on a team of athletes (approximately 25 athletes) in under 3 hours. We utilized multiple stations that included separate check-in, anthropometrics, marker placement, instruction, and data collection. This provided an opportunity for a large number of personnel to be appropriately trained in a few key areas instead of a few personnel taking on multiple responsibilities. Data collection forms with trial names and randomization order were produced prior to the data collection session and populated via scripting techniques within the motion capture software.

A two-step process was utilized to check the quality of the marker coordinates and to determine proper tracking identification. First, a technician from each laboratory performed the quality control of each trial detailing any potential errors within a spreadsheet. Potential errors, such as marker misidentification and small gaps, were immediately fixed. Secondly, a senior researcher reviewed the trials and addressed any additional concerns with the data. Gaps in marker position data of less than 10% of the marker sampling frequency (Sites A and B ≤24 frames; Site C ≤20 frames) were interpolated using a cubic spline fill. Due to the inherent high hip and trunk flexion in combination with clothing, small 9mm markers, and the rapid rate of data collection, larger gaps in ASIS marker position data were present in a number of subjects. Based on the results of this study, we have now modified camera placement and include redundant pelvis markers. Gaps in ASIS marker position data greater than 10% but less than 25% of the marker sampling frequency were interpolated using a virtual marker fill based on the fixed relative distance from the contralateral ASIS marker and hip joint center and the sacrum. Our lab has internally validated this approach and verified that it creates an acceptable amount of root mean square error when joint angle curves are compared between real and virtual filled ASIS marker data. It also creates no clinically relevant differences in peak computed joint angles.

Motion data were subsequently processed through Visual 3D (v4.86, C-motion Inc. Germantown, MD, USA) using batch scripts via the motion capture software and Matlab (vR2012b, Mathworks Inc. Natick, MA, USA). All marker and force data were subsequently filtered using a low-pass fourth-order Butterworth filter, sampling at 12 Hz, and post-processed using the same custom Visual 3D and Matlab coding to compute lower extremity joint Euler angles. Force data and computed kinematic data were used to compute joint moments. All joint kinematic and kinetics data from each trial were exported from Visual 3D and plotted as an additional quality control step. We have found this identifies a small percentage of erroneous data from incorrect model based analyses that would not be identified when batch processing large amounts of data.

Peak hip, knee, and ankle angles and moments were calculated during stance from the DVJ using Visual 3D. Stance was defined as the time period between initial contact and take-off (i.e. when the vertical ground reaction force (GRF) exceeded 10N and subsequently fell below 10 N). The average of three trials was used in the current analysis. A two-way, random-effects model of intraclass correlation coefficients (ICC) were used to compute the reliability of peak angles and moments from trial-to-trial at each site (3,k), between each combination of sites (3,1), and among all three sites (3,k). ICC values were classified as ICC>0.75 = excellent, 0.4≤ICC≤0.75 = good, or ICC<0.4 = poor^7^. Coefficients of multiple correlation (CMC) were also computed using the methods described by Kadaba et al.^8^ to measure the variability of joint angle and moment waveforms during stance at each site, between each combination of sites, and among all three sites^8^. Standard error of the measurement (SEM) for each peak kinematic and kinetic variable was also computed and reported. Subject demographic information was compared across sites using an Analysis of Variance with one repeated measure. A priori significance was set at α<0.05.

## Results

The average (± standard deviation) age of subjects at the time of the first testing session (Site A) was 

15.3±1.0 years. The average height and mass at the same time point were 169.3±4.5 cm and 62.3±6.8 kg, respectively. There were no significant changes in height (p=0.248) or body mass (p=0.096) during the study period.

### Kinematics

The reliability of peak kinematic variables among all three sites was rated as excellent (Range: 0.762-0.893; Table 2). There was greater variability in waveforms among all three sites, and CMCs ranged from 0.456 to 0.954. All sagittal-plane waveforms had CMCs >0.9, whereas none of the coronal- or transverse-plane waveforms had CMCs greater than 0.7. 

**Table 2.** Between-site reliability for peak kinematic and kinetic variables.

	**AMONG SITES**		**Site A - Site B**			**Site B - Site C**			**Site A - Site C**							
	**CMC**	**ICC (3,k)**	**CMC**	**ICC (3,1)**	**SEM**	**CMC**	**ICC (3,1)**	**SEM**	**CMC**	**ICC (3,1)**	**SEM**
**Joint Angles(** **^o^** **)**											
Hip flexion	0.932±0.09	0.860	0.934±0.07	0.536	5.0	0.974±0.02	0.765	3.4	0.955±0.04	0.719	4.2
Hip adduction	0.456±0.31	0.855	0.644±0.28	0.724	2.3	0.709±0.25	0.700	2.1	0.638±0.28	0.573	2.8
Hip internal rotation	0.693±0.13	0.843	0.823±0.14	0.620	4.0	0.847±0.09	0.673	4.1	0.813±0.11	0.629	4.7
Knee flexion	0.945±0.10	0.832	0.944±0.10	0.550	4.7	0.981±0.02	0.525	4.5	0.962±0.07	0.762	3.7
Knee abduction	0.578±0.24	0.893	0.811±0.11	0.715	2.2	0.795±0.23	0.758	2.2	0.810±0.17	0.728	2.3
Knee internal rotation	0.523±0.20	0.860	0.827±0.15	0.735	2.9	0.806±0.16	0.547	4.0	0.830±0.13	0.741	2.9
Ankle dorsiflexion	0.954±0.05	0.762	0.948±0.09	0.789	2.0	0.960±0.08	0.373	3.4	0.969±0.03	0.451	3.5
Ankle Eversion	0.608±0.21	0.777	0.756±0.23	0.470	2.7	0.804±0.16	0.655	1.9	0.786±0.21	0.711	1.7
											
Joint Moments (N*m*kg **^-1^** **)**											
Hip flexion	0.862±0.14	0.830	0.851±0.14	0.660	0.18	0.917±0.04	0.550	0.22	0.886±0.09	0.673	0.15
Hip adduction	0.643±0.13	0.626	0.722±0.16	0.553	0.10	0.780±0.12	0.140	0.13	0.697±0.12	0.384	0.10
Hip internal rotation	0.700±0.14	0.545	0.775±0.15	0.605	0.07	0.775±0.12	0.293	0.12	0.712±0.16	0.140	0.14
Knee flexion	0.818±0.22	0.789	0.860±0.13	0.385	0.22	0.936±0.04	0.734	0.16	0.872±0.12	0.504	0.18
Knee abduction	0.615±0.09	0.620	0.729±0.14	0.216	0.11	0.847±0.12	0.431	0.13	0.765±0.12	0.342	0.13
Knee internal rotation	0.493±0.13	0.692	0.738±0.14	0.344	0.06	0.690±0.19	0.370	0.07	0.729±0.18	0.612	0.04
Ankle dorsiflexion	0.743±0.22	0.705	0.799±0.15	0.526	0.13	0.916±0.04	0.639	0.09	0.819±0.13	0.223	0.16
Ankle Eversion	0.540±0.21	-0.112	0.706±0.19	0.798	0.05	0.673±0.22	-0.288	0.15	0.592±0.21	-0.250	0.15

CMC: coefficient of multiple correlation; ICC: intra-class correlation coefficient (mean +/- standard deviation); SEM: standard error of the measurement.

Between sites, there were only four instances in which the reliability of peak kinematics variables was rated as excellent (Table 2). The majority of peak variables were rated as good. Peak ankle dorsiflexion between sites A and B was the only kinematic variable rated as poor; however, it had the greatest CMC value among all between-site comparisons. 

Within each site, the reliability of peak kinematic variables from trial-to-trial was consistently rated as excellent. CMCs varied from 0.526 to 0.991 (Table 3). CMCs for each variable were greater in the sagittal plane than the coronal and transverse planes. 

**Table 3.** Within-site, trial-to-trial reliability.

	**Site A**			**Site B**			**Site C**		
	**CMC**	**ICC (3,k)**	**SEM**	**CMC**	**ICC (3,k)**	**SEM**	**CMC**	**ICC (3,k)**	**SEM**
Joint Angles^o^									
Hip flexion	0.974±0.02	0.908	2.6	0.979±0.01	0.900	1.9	0.982±0.02	0.947	1.7
Hip adduction	0.610±0.30	0.950	0.9	0.672±0.25	0.916	1.3	0.526±0.35	0.896	1.5
Hip internal rotation	0.813±0.19	0.963	0.7	0.830±0.13	0.920	1.2	0.818±0.17	0.946	0.7
Knee flexion	0.987±0.10	0.932	2.3	0.991±0.01	0.910	2.0	0.991±0.01	0.925	2.3
Knee abduction	0.816±0.15	0.989	0.5	0.828±0.10	0.973	0.7	0.896±0.08	0.987	0.5
Knee internal rotation	0.721±0.18	0.983	0.9	0.825±0.07	0.983	0.8	0.725±0.14	0.977	1.3
Ankle dorsiflexion	0.987±0.01	0.947	1.2	0.942±0.22	0.942	1.4	0.989±0.01	0.928	1.7
Ankle Eversion	0.801±0.18	0.971	0.8	0.684±0.26	0.883	1.4	0.766±0.15	0.923	1.1
									
Joint Moments (N*m*kg **^-1^** **)**									
Hip flexion	0.851±0.26	0.806	0.07	0.933±0.05	0.784	0.09	0.935±0.04	0.896	0.05
Hip adduction	0.773±0.09	0.766	0.04	0.776±0.15	0.628	0.06	0.785±0.12	0.675	0.07
Hip internal rotation	0.811±0.16	0.833	0.03	0.896±0.07	0.827	0.03	0.852±0.19	0.856	0.04
Knee flexion	0.889±0.16	0.741	0.09	0.932±0.06	0.900	0.08	0.917±0.05	0.882	0.07
Knee abduction	0.667±0.23	0.849	0.04	0.747±0.24	0.910	0.04	0.770±0.19	0.881	0.03
Knee internal rotation	0.579±0.24	0.827	0.03	0.593±0.20	0.603	0.05	0.542±0.27	0.793	0.03
Ankle dorsiflexion	0.774±0.16	0.897	0.02	0.884±0.08	0.803	0.03	0.893±0.06	0.820	0.02
Ankle Eversion	0.658±0.29	0.899	0.05	0.847±0.10	0.951	0.03	0.825±0.08	0.496	0.08

CMC: coefficient of multiple correlation; ICC: intra-class correlation coefficient (mean +/- standard deviation); SEM: standard error of the measurement.

### Kinetics

The reliability of peak kinetic variables among sites varied greatly (range: -0.112-0.830) (Table 2). ICCs were greatest in the sagittal plane for each variable. CMC values varied from 0.493 to 0.862 and were consistently greatest in the sagittal plane for each variable. 

Between sites, there was only one instance in which ICCs were rated as excellent. The remaining comparisons were rated as good or poor. Kinetic waveforms for each variable had CMC values that were either similar or slightly less than the respective kinematic waveforms.

Within each site, the reliability of peak kinetic variables from trial-to-trial was consistently rated as excellent with only five instances in which reliability was rated as good (Table 3). The kinetic waveforms had CMC values similar to their respective kinematic comparisons. 

## Discussion

In general, the kinematic data were reliable and reproducible for use in large multicenter trials. However, the reliability of the kinetic data did not appear to be high, hence, this data may be best fit for use at individual sites to train individuals. This kinetic data may also be useful for biofeedback training. For instance, feedback regarding position and technique during sports-related movements may increase an athlete's awareness and allow him or her to make adjustments during training. Using real-time kinematic biofeedback may provide an intriguing option for delivering augmented feedback and could maximize the effectiveness of traditional neuromuscular intervention programs. Recent studies with real-time gait retraining have reinforced the concept of providing critical feedback with detailed real-time motion analysis data^9^^-^ 11. Multiple studies support the idea that real-time feedback modified potential risk factors related to different knee pathologies. Both immediate and long-term improvements have been identified^9^^-^ 11.

### Challenges of Multicenter BiomechanicalEpidemiologic Studies

#### Subject Recruiting

We have faced and met many challenges recruiting subjects (i.e. the athletes) in school systems, but to a greater extent at the junior high school level than the high school level in the county school systems. For example, some of the county junior high schools do not have organized sports teams. We addressed this challenge by recruiting teams from recreational leagues and parochial (religious) schools within the county and adjoining counties in order to capture those athletes who go on to play high school sports within the school system.

#### Data Quality Control

Teams at all sites completed their data processing pipeline for the biomechanical analyses. With data collected and processed over multiple sites, it was found that a small percentage of movement trials had incorrectly tracked markers, even though all trials had been inspected by a human operator. Due to the high volume of data, it was not possible to do a more thorough inspection of the raw data; therefore, two data verification steps were added to the processing pipeline. The first step involved a complete tracking quality control step at the home site. The second step processed the marker trajectories using a simplified skeleton model with a few degrees of freedom. When presented with incorrectly tracked marker coordinates, this analysis reported an error because the simplified skeleton was unable to fit the data. Trials where this error was detected were sent back to the human operator for re-tracking, and the others were processed further into joint kinematics and kinetics. A third quality control step was added at the end of the biomechanics pipeline. A human operator looked at groups of curves representing the main biomechanical variables, each curve representing one trial. Outlying curves were identified and the marker tracking of those trials was re-examined for correctness. If correct, the data was used for further statistical analysis. If not, marker tracking was corrected or, if the data was corrupted or incomplete, the trial was discarded. The authors are currently developing a fourth quality control step using a confidence interval-based approach for automatic detection of outliers.

#### Peak versus Mean Variable Values

Our research group normally reports both peak and mean variable values across the entire stance phase in our studies^12^. We attempt to mitigate the effects of potential moment artifacts by reporting the peak values averaged across three trials per subject. For example, peak KAM occurs approximately 50ms after initial contact during a run-cut, a time at which joint moment artifacts are likely to occur. Conversely, peak KAM during a DVJ does not always occur soon after initial contact when large artifacts are likely to occur. Considering the stance time of a typical DVJ is approximately 400 msec, the peak KAM would occur closer to 100ms and therefore not located where impact artifacts occur during a run-cut^13^.

#### Data Filtering

How one decides to filter and analyze motion data is both an art and a science that requires careful consideration of both the tasks being analyzed and the outcome variables of interest. For these reasons, there are several subtleties and some possible flaws in other studies that warrant clarification. Our research team understands the benefits of testing the reliability of kinematic and kinetic data at matched cutoff frequencies and we have been filtering our motion data at matched frequencies for several years^1^^,^ 14 ^-^ 16. However, universally dismissing studies that use unmatched cutoff frequencies or suggesting that earlier conclusions should be reconsidered - specifically, those from our 2005 study^12^ - is unfounded. We must not fail to acknowledge the power of the prospective case-cohort design. Principally, prospective designs prevent investigators from potentially biasing their cohorts because they prospectively treat the data uniformly for all of their subjects: those who eventually go on to suffer an injury and those who did not. Thus, if properly designed, prospective cohort data will result in valid and reliable findings.

The effects of filtering may render measured biomechanical variables less reliable as injury prediction tool than previously thought. This is why it is important that authors report the reliability of their data. If investigators do not report the reliability of their measures in their study paper or elsewhere in the literature, their conclusions should be interpreted with caution.

#### Data Limitations-Differences in Movement tasks-validity, reliability issues

It is likely incorrect for investigators to assume that differences in kinematic and kinetic (moment) calculations for one movement directly relate to all other movements that involve high-impact accelerations. For example, it has been shown that relative loads may vary greatly between a drop vertical jump (DVJ) and a cutting movement. All movement tasks that are subject to large forces and accelerations fall victim to a certain degree of specificity of angles, loads, and artifacts. For example, a run-cut task is subject to much larger frontal-plane forces and segment accelerations than a DVJ task; therefore, Knee Abduction Moment (KAM) measured during a run-cut task is likely more sensitive to cutoff frequency than KAM measured during a DVJ. However, large artifacts are typically reserved for the planes of motion in which these large forces and accelerations occur. For example, Kristianslund et al.^17^ reported a mean peak KAM between 75-150 Nm during a run-cut task, whereas we reported mean peak KAM between 15-45 Nm during a DVJ^17^. We also previously compared a DVJ to a jump stop side-cut movement and reported significant differences in KAM and Knee Abduction Angle (KAA) between the two movements^16^. An analysis of our most recent DVJ data indicate that filtering frequency may have only a small effect on the magnitude of peak KAM, and a negligible effect on the relative ranking of subjects based on peak KAM^18^.

#### Validity of Conclusions and Interpretation of Findings

In order to conduct a proper measurement of the validity of investigators' conclusions from a coupled biomechanical-epidemiologic cohort prospective trial, one would need to examine the fidelity with which injuries were prospectively tracked before a study of any task can be effectively evaluated for injury risk assessment. There are also many potential bias problems introduced in poorly designed cohort and intervention trials. Potential biases abound such as selection bias, reporting bias, and absence of blinding. For example, in a recent study published as a Level One trial in The American Journal of Sports Medicine, significant limitations in the design of the study may have affected the results and their interpretation^19^. Each coach and all of the athletes knew whether or not they had been assigned to the intervention program, and all were well aware of the expected outcomes of using the program, which had a track record of reducing injuries. This knowledge could potentially have led to a placebo effect among players using the intervention - there was no placebo or "sham" treatment to blind the researchers or study subjects. In addition, the players on the teams that did not use the intervention were older (almost two years older on average), taller, and heavier than the athletes on the teams that took part in the program. One would expect more injuries in bigger, taller athletes independent of the intervention. At the most basic level, simple physics apply - the bigger the study subject is, the harder he or she will fall.

### Injury tracking - an important effector of validity of follow-up

In order to conduct a proper measurement of the validity of our findings and conclusions, one would need to track injuries prospectively before a run-cut task could be effectively used for injury risk assessment. Many studies are not designed properly to answer the questions upon which they speculate. A properly designed study requires an approach that includes apples-to-apples comparisons of groups and to other studies using identical data collection, reduction techniques, injury tracking methods, and analyses.

Replication of any study is important for its tenets to gain widespread acceptability. ACL-injury risk factors have proven to be complex and multifaceted with mechanical, biological, hormonal, and psychosocial components. KAM and KAA are certainly prominent, predictive markers for ACL injury risk, and have been repeatedly validated^10^^,^ 20 ^-^ 23, but are only two of many important factors. We have new data that indicates that KAA may be as strong a predictor as KAM. These data are important as we move forward with our secondary kinematic two-dimensional analyses and develop more comprehensive and generalizable clinic-based predictive models.

### Significance of coupled biomechanicalepidemiologic study findings

The findings of these coupled biomechanicalepidemiologic studies should provide a foundation for approaching both the mechanistic questions underlying injury risk disparities between individuals and groups, such as sexes, as well as increase our ability to direct high-risk athletes to effective, neuromuscular interventions targeted at specific, measured deficits related to pubertal growth and development.

We performed these parallel studies at the three sites all within two weeks of one another. The goals of these studies were to develop the reliability across sites in a large cohort in order to conduct large multicenter randomized controlled trials. We tested and cross-validated three different biodynamics laboratories (at OSU, CCH, and UK) to collect data on the same medium-sized cohort of subjects. This resulted in adequate statistical power and allowed us to examine injury events as both secondary and primary outcomes. Though KAA and KAM are prominent markers for ACL injury risk and have been demonstrated repeatedly to predict increased ACL injury risk^12^^,^ 24, but are only two of many potentially important factors. We have new data that indicates that knee abduction angle may be as strong a predictor as KAM. This is important as we move forward with our secondary kinematic 2D analyses and development of more comprehensive and generalizable clinic-based predictive models.

## Summary and conclusions

This extended study method developed with a multi-institutional, multidisciplinary team will likely yield more robust results with increased generalizability and applicability to diverse populations. The additional analyses will provide a foundation for addressing important mechanistic questions; however, they are extremely costly and time-consuming and require assistance. Nevertheless, the added approaches proposed in this supplement will foster the development of a clinician-friendly assessment tool that will enhance the translation of the study results into use in the medical community.

We suggest future collaborative, multicenter, multi-institutional studies that include apples-to-apples comparison of data grouped across sites using identical data collection, reduction techniques, injury tracking methods, and analyses. Replication of any study is important for establishing widespread acceptability. Injury risk factors have proven to be a complex, multifaceted problem with biological, hormonal, mechanical, and psychosocial factors. For example, KAM is certainly a prominent marker for ACL injury risk and has been demonstrated repeatedly, but it is only one of many important factors. We have new data that indicates that knee abduction angle may be as strong a predictor as KAM. This is important as we move forward with our secondary kinematic 2D analyses and developing more comprehensive and generalizable clinic-based predictive models.

### Future directions and plans

Our research consortium continues to utilize a prospective longitudinal design for school-sponsored soccer and basketball teams from multiple school systems, which are recruited, tested, and tracked. Female and male subjects from high schools and junior high schools are being screened prior to the start of each consecutive soccer, volleyball, and basketball seasons. We have tested the basketball players for several consecutive years. We have previously tested and measured the longitudinal reliability and validity of all of the data collected during testing from one site at CCH^1^. The biomechanics and sports medicine research communities should continue to utilize these analyses to evaluate both the pre-test profiles as well as to determine the effects of prospective randomized controlled trial study designs. We will conduct sports injury surveillance on all of the athletes for two consecutive years following the athletes' enrollment into the study.
